# Prospective Application of Ferroptosis in Hypoxic Cells for Tumor Radiotherapy

**DOI:** 10.3390/antiox11050921

**Published:** 2022-05-07

**Authors:** Jing Su, Qin Zhao, Zhuangzhuang Zheng, Huanhuan Wang, Chenbin Bian, Lingbin Meng, Ying Xin, Xin Jiang

**Affiliations:** 1 Jilin Provincial Key Laboratory of Radiation Oncology & Therapy, The First Hospital of Jilin University, Changchun 130021, China; jingsu21@mails.jlu.edu.cn (J.S.); jluzhaoqin09@jlu.edu.cn (Q.Z.); zhengzz2715@mails.jlu.edu.cn (Z.Z.); wanghh2714@mails.jlu.edu.cn (H.W.); biancb21@mails.jlu.edu.cn (C.B.); 2 Department of Radiation Oncology, The First Hospital of Jilin University, Changchun 130021, China; 3 NHC Key Laboratory of Radiobiology, School of Public Health, Jilin University, Changchun 130021, China; 4 Department of Hematology and Medical Oncology, Moffitt Cancer Center, Tampa, FL 33612, USA; lingbin.meng@moffitt.org; 5 Key Laboratory of Pathobiology, Ministry of Education, Jilin University, Changchun 130021, China

**Keywords:** hypoxia, ferroptosis, reactive oxygen species (ROS), lipid peroxidation, radiotherapy, Nrf-2, AMPK

## Abstract

Radiation therapy plays an increasingly important role in cancer treatment. It can inhibit the progression of various cancers through radiation-induced DNA breakage and reactive oxygen species (ROS) overload. Unfortunately, solid tumors, such as breast and lung cancer, often develop a hypoxic microenvironment due to insufficient blood supply and rapid tumor proliferation, thereby affecting the effectiveness of radiation therapy. Restraining hypoxia and improving the curative effect of radiotherapy have become difficult problems. Ferroptosis is a new type of cell death caused by lipid peroxidation due to iron metabolism disorders and ROS accumulation. It plays an important role in both hypoxia and radiotherapy and can enhance the radiosensitivity of hypoxic tumor cells by amplifying oxidative stress or inhibiting antioxidant regulation. In this review, we summarize the internal relationship and related mechanisms between ferroptosis and hypoxia, thus exploring the possibility of inducing ferroptosis to improve the prognosis of hypoxic tumors.

## 1. Introduction

Cancer is one of the most harmful diseases to human health. Due to the progress of treatment technology, the popularization of early cancer screening and other modalities has led to a year-by-year decline in cancer mortality. The latest statistics show a 31% decrease in total cancer deaths between 1991 and 2018 [1]. Radiotherapy (RT) can inhibit the progression of various cancers through radiation-induced DNA breakage and reactive oxygen species (ROS) overload [2]. Unfortunately, solid tumors such as breast cancer and lung cancer often develop a hypoxic microenvironment due to insufficient blood supply and rapid tumor proliferation, thereby affecting the effectiveness of RT. Radiation resistance caused by hypoxia often fails to achieve the expected outcome in the treatment of solid tumors [3]. Overcoming resistance caused by hypoxia has always been a concern for the general population.

Ferroptosis is a new type of cell death caused by lipid peroxidation due to iron metabolism disorders and ROS accumulation. It is different from other regulated cell death (RCD), such as apoptosis, autophagy and pyroptosis, in morphology and mechanisms. Morphologically, ferroptosis is neither featured with typical apoptotic features, such as chromatin agglutination and apoptotic body formation, nor the formation of autophagic vacuoles, unique autophagy features or the release of proinflammatory factors, which is a typical feature of pyroptosis; instead, ferroptotic cells mainly manifest as shrunken mitochondria with increased membrane density and disappeared mitochondrial cristae [4,5]. Mechanistically, although other RCDs can also be triggered by ROS overload [6], ferroptosis is always accompanied by significant iron accumulation, glutathione (GSH) depletion, and changes in genes related to iron homeostasis and lipid metabolism [7]. Polyunsaturated fatty acid (PUFA) is the source of ferroptosis. PUFAs participate in the synthesis of membrane phospholipids catalyzed by acyl-CoA synthetase long-chain family member 4 (ACSL4) and lysophosphatidylcholine acyltransferase 3 (LPCAT3) [8,9], then cytochrome P450 oxidoreductase (POR) and lipoxygenases (LOX) will oxidize polyunsaturated phospholipids (PUFA-PL) to lipid peroxides (L-OOH) [10,11,12]. Normally, on the one hand, the cytotoxic L-OOH can be reduced to the corresponding alcohols by glutathione peroxidase (GPX4) [13]. On the other hand, it can be converted into L-OO^.^ through Fenton reaction, while L-OO^.^ can be reduced by ubiquinol (CoQH2) to protect cells from lipid peroxidation [14,15]. However, when Fe^2+^ or PUFA is overloaded or the antioxidant system is imbalanced, L-OO^.^ will accumulate in large quantities, resulting in lipid peroxidation of membrane phospholipids, and ultimately lead to ferroptosis [7]. Cystine/glutamate antiporter solute carrier family 7 member 11 (SLC7A11; also known as xCT) is a key factor regulating ferroptosis. It controls the synthesis of GSH, which allows GPX4 to reduce L-OOH [16]. Furthermore, tetrahydrobiopterin (BH4) and ferroptosis suppressor protein 1 (FSP1) also play an important role in inhibiting ferroptosis by producing CoQH2 [14,17]. In addition, with the increasing research on ferroptosis, it has been found that it plays an important role in the process of cell death caused by radiation. Lang et al. found that radiation can activate the ataxia-telangiectasia mutated gene (ATM) and inhibit the expression of SLC7A11, thereby promoting ferroptosis [18]. Moreover, Lei et al. verified this view and found that ionizing radiation (IR) significantly increased the staining of C11-BODIPY and 4-hydroxynonenal (4-HNE) in tumor cells, thus causing typical morphological changes in ferroptosis [19]. Further studies have shown that ferroptosis inducers such as sulfasalazine, ras-selective lethal small molecule 3 (RSL-3), and cyst(e)inases have an obvious effect on radiation sensitization; such sensitization is mainly related to ROS accumulation and lipid peroxidation [18].

The radiation resistance of hypoxic tumor cells is closely related to antioxidant stimulation. For example, hypoxia-inducible factor 1 (HIF-1) enhances the activities of glycolysis, serine synthesis, and pentose phosphate pathways. In addition, it increases the production of antioxidants, thus buffering radiation-induced ROS and resulting in radiation resistance. In addition, hypoxia itself increases the production of ROS, triggering a feedback loop and stimulating a metabolic process conducive for generating antioxidants; it can activate autophagy to accelerate the removal of ROS products, thus making cells radiation-resistant [3,20]. However, due to the large accumulation of ROS, hypoxic tumor cells rely heavily on the antioxidant system to maintain homeostasis. As a result, they become extremely sensitive to oxidative damage. Studies have shown that ferroptosis can be induced by inhibiting GSH synthesis and destroying the redox balance, thus improving the radiosensitivity of hypoxic tumor cells [21]. We believe that ferroptosis can enhance the radiosensitivity of hypoxic tumor cells by amplifying oxidative stress or inhibiting antioxidant regulation, providing a new approach for the treatment of solid tumors. Therefore, we summarize the internal relationship and possible regulatory mechanism between ferroptosis and hypoxia, as well as explore the possibility of inducing ferroptosis to improve the prognosis of hypoxic tumors.

## 2. Hypoxia Protects Tumor Cells from Ferroptosis by Regulating Iron Homeostasis

Hypoxia has been recognized as one of the basic and important characteristics of solid tumors; it plays an important role in various physiological events, such as cell proliferation, angiogenesis, metabolism, tumor invasion, and metastasis [22]. HIF-1 is a key regulator of hypoxia response in tumor cells and plays a key role in the adaptation of tumor cells to the hypoxic microenvironment. Hypoxia and HIF-1 overexpression are associated with radiation and chemotherapy resistance, as well as poor clinical prognosis of solid tumors [22,23]. Radiation resistance caused by hypoxia is considered to be the main reason why radiation therapy for solid tumors fails to achieve the expected results. Different mechanisms have been proposed to explain radiation resistance caused by hypoxia, among which the oxygen fixation hypothesis is the most accepted [24]. In addition, the upregulation of HIF-1α and enhancement of antioxidant system activity caused by hypoxia are also considered to be important factors for radiation resistance [3,20]. Interestingly, recent studies have shown that hypoxia can protect tumor cells from ferroptosis [25], which may explain the poor prognosis of hypoxic tumors.

**Figure 1:**
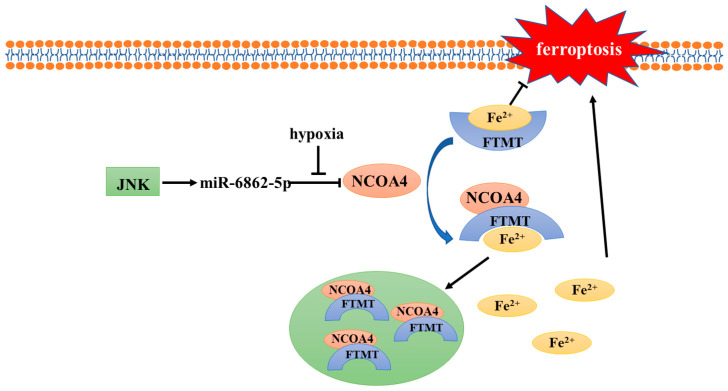
Mechanism of NCOA4 regulation under hypoxia.

In the body, cellular iron is essential for maintaining various metabolic pathways. Excessive free iron (Fe^3+^ or Fe^2+^) may lead to oxidative damage and cell death [26]. In addition, intracellular free iron can induce ferroptosis by activating arachidonate lipoxygenases (ALOXs) and promoting ROS production [4,27]. Therefore, the maintenance of iron homeostasis plays a crucial role in cell development; ferritin plays the most important role in regulating intracellular iron homeostasis. Ferritin heavy chain (FTH), ferritin light chain (FTL), and mitochondrial ferritin (FTMT) are three known ferritin proteins that have ferroxidase activity which can store Fe^3+^ and reduce intracellular free Fe^2+^ levels [28], thus playing an important role in ferroptosis. The release of iron in ferritin is regulated by the process of ferritinophagy; nuclear receptor coactivator 4 (NCOA4), its main regulator, directly binds to FTH and transports the complex to the lysosome for degradation [29]. The results of Fuhrmann et al. demonstrated that under hypoxic conditions, the transcription rate of NCOA4 decreases, leading to an increased FTH and FTMT expression, which protects cells from ferroptosis [25] (Figure 1). Subsequently, this was verified in diabetic mice, osteoporotic mice, and other animal models [30,31]. Furthermore, it was proven that the downregulation of NCOA4 and inhibition of ferroptosis under hypoxic conditions were associated with poor prognosis of stomach adenocarcinoma and hepatocellular carcinoma [32,33]. In conclusion, hypoxia represses ferritinophagy-mediated ferroptosis and leads to unfavorable prognosis; correspondingly, the induction of ferritinophagy and disruption of iron homeostasis may be an effective way to improve the therapeutic effect of hypoxic tumor cells.

The transcription of NCOA4 is related to miR-6862-5p. Under normoxic conditions, NCOA4 can bind with FTMT and Fe^2+^, and then send the complex to the autolysosome for degradation. However, the transcription of NCOA4 is inhibited when hypoxia occurs, thus blocking ferritinophagy and inhibiting ferroptosis. JNK, c-jun N-terminal kinase; NCOA4, nuclear receptor coactivator 4; FTMT, mitochondrial ferritin.

## 3. Bidirectional Regulation of Hypoxia-Inducible Factors on Ferroptosis

Hypoxia-inducible factors (HIFs) are ubiquitous in human and mammalian cells and can be stably expressed under hypoxic conditions. HIFs are heterodimeric transcription factors, composed of α and β subunits. The activity of HIFs is mainly dependent on α subunits, while β subunits are responsible for maintaining its stability [34]. The reason why α and β subunits function differently is that α subunits have an oxygen-dependent degradation domain (ODDD) in their structures [35]. In addition to ODDD, the α subunits have two transactivation domains: NH2-terminal (N-TAD) and COOH-terminal (C-TAD). They are responsible for the transcriptional activity of HIF-1/2α [36]. There are three subtypes of HIF-α: HIF-1α, HIF-2α, and HIF-3α [37,38]. HIF-1α and HIF-2α are particularly important for hypoxia response and can form complexes with HIF-1β [39]. The role of HIF-3α is unclear, but it has been suggested that HIF-3α may be involved in the transcriptional inhibition of HIF-1α [40].

The regulatory pathways of HIF-1/2α can be divided into oxygen-dependent and oxygen-independent pathways. Under normoxic conditions, proline residues (P402 and P564) within the ODDD of HIF-1α subunits and (P405 and P531) of HIF-2α subunits are hydroxylated by prolyl hydroxylases domain enzymes (PHD1-4) [41,42]. Hydroxylated HIF-1/2α can be recognized and ubiquitinated by the von Hippel–Lindau protein (pVHL), which mediates the assembly of the VHL/elongin C/elongin B/cullin-2 E3 ubiquitin ligase complex and is subsequently degraded by proteasomes [43]. In addition to prolyl hydroxylase, factor inhibiting HIF (FIH) is another cellular dioxygenase which could hydroxylate the 803 and 847 residues of asparagine within the C-TADs of HIF-1α and HIF-2α, respectively, and inhibit the transcription of HIF-1/2α [44]. However, PHDs and FIH are both oxygen dependent. Under hypoxic conditions, their activity is inhibited, allowing HIF-1/2α to escape ubiquitination and be transported to the nucleus after binding with HIF-1β [45,46]. In addition, ROS can also regulate the activity of PHDs and FIH. Studies have shown that NADPH oxidase 1 (NOX1) and NADPH oxidase 4 (NOX4) maintain HIF-2α expression in renal carcinoma through ROS generation, contributing to renal carcinogenesis [47]. Moreover, in recent years, several studies have shown that HIF can be regulated without oxygen; for example, heat shock protein 90 (Hsp90) can bind to HIF-1/2α subunits to inhibit their degradation [48], PI3K/AKT and MAPK/ERK pathways participate in the regulation of HIF-1/2α expression [49,50], and post-transcriptional acetylation and deacetylation regulate HIF-1/2α transcription and stability [51,52].

**Figure 2:**
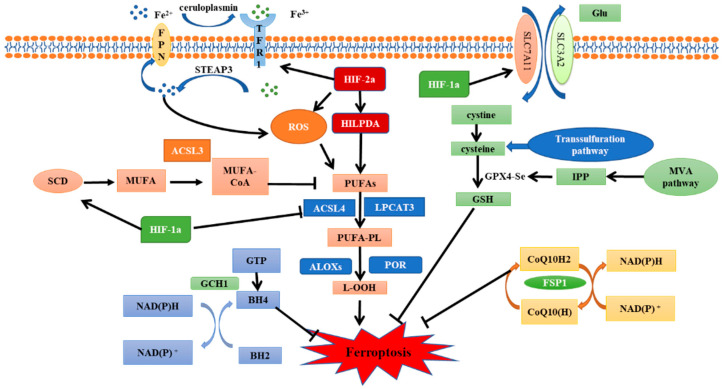
The regulatory pathways of ferroptosis and the role of HIF.

HIF-1/2α play a crucial role in the response of tumors to low oxygen [53]. Furthermore, the expression of HIF-1/2α is often associated with poor prognosis and chemoradiotherapy resistance [54]. Therefore, inhibiting HIF-1/2α expression and their downstream pathways activation is considered to be a reliable way to enhance radiosensitivity. Recently, HIF-1/2α subunits were found to play an important role in the regulation of ferroptosis (Figure 2), which further proved that ferroptosis inhibition is an important factor leading to unfavorable prognosis of hypoxic tumors.

Ferroptosis is regulated by iron metabolism, lipid metabolism and antioxidant system. SLC7A11-GPX4-GSH pathway, FSP1-CoQ10-NAD (P)H pathway and GCH1-BH4-DHFR pathway are several classic ferroptosis inhibition pathways. HIF-1α has a significant inhibitory effect on ferroptosis. It can promote the expression of SCD1 to increase the synthesis of MUFA, inhibit the expression of ACSL4 to avoid lipid peroxidation, and reduce the degradation of SLC7A11 to enhance the antioxidant capacity of cells. In contrast, HIF-2α promotes ferroptosis. Activation of the HIF-2α-HILPDA pathway can promote the synthesis of PUFAs to induce lipid peroxidation. HIF-2α can also regulate iron metabolism gene to increase intracellular free iron content and oxidize cysteine to increase ROS production. SLC7A11, Cystine/glutamate antiporter solute carrier family 7 member 11; GPX4, glutathione peroxidase 4; GSH, glutathione; FSP1, ferroptosis suppressor protein 1; GGH1, GTP Cyclohydrolase 1; BH4, tetrahydrobiopterin; DHFR, dihydrofolate reductase; HIF, hypoxia-inducible factor; SCD1, stearoyl-CoA desaturase 1; MUFA, monounsaturated fatty acids; ACSL4, acyl-CoA synthetase long-chain family member 4; HILPDA, hypoxia inducible lipid droplet-associated proteins; and PUFAs, polyunsaturated fatty acids.

### 3.1. HIF-1α—A Negative Regulator of Ferroptosis

HIF-1α plays an important role in the metabolic regulation of hypoxic cells. Activation of HIF-1α can enhance the activities of glycolysis, serine synthesis, and pentose phosphate pathways [55], thereby allowing cells to adapt to the hypoxic environment. The massive production of antioxidants induced by HIF-1α is also considered as one of the mechanisms of radiation resistance in hypoxic cells. In recent years, it has been found that HIF-1α expression is associated with ferroptosis.

To begin with, HIF-1α can inhibit ferroptosis by regulating SLC7A11. SLC7A11 expression is positively associated with HIF-1α in human hepatocellular carcinoma (HCC) tissues [56]. It has been shown that HIF-1α can inhibit methyltransferase-like 14 (METTL14) expression, thus inhibiting the degradation of SLC7A11 mRNA and reducing the sensitivity of HCC cells to ferroptosis [57]. Moreover, the HIF-1α/SLC7A11 pathway has also been reported in the nervous system and oral squamous cell carcinoma (OSCC) [58,59,60]. Unfortunately, one study found that HIF-1α does not regulate SLC7A11 in breast cancer cells [61]. Therefore, the application scope and specific mechanism of this pathway need to be further studied. HIF-1α can also inhibit ferroptosis by regulating lipid metabolism. Yang et al. demonstrated in a variety of human tumor cell lines that aryl hydrocarbon receptor nuclear translocator-like protein 1 (ARNTL, also call BMAL1) inhibits ferroptosis by inhibiting EGLN2 (also call PHD2) activation and activating HIF-1α. Additionally, HIF-1α-induced ferroptosis inhibition may be related to its role in promoting lipid storage [62]. Conversely, EGLN1 (also call PHD1) inhibits ferroptosis by promoting lymphoid-specific helicase (LSH) expression in lung cancer cell lines [63]. This result further proves that the regulation of HIF-1α on ferroptosis is complex and variable. Furthermore, ACSL4 is a key enzyme in the formation of polyunsaturated fatty acids; HIF-1α inhibits the synthesis of ACSL4 to inhibit ferroptosis [64]. In nasopharyngeal carcinoma (NPC), HIF-1α upregulates the expression of stearoyl-CoA desaturase 1 (SCD1), which mediates the production of monounsaturated fatty acids and inhibits ferroptosis [65]. Finally, a study of cervical cancer (CC) demonstrated that HIF-1α activation can suppress ferroptosis by destroying iron homeostasis imbalance [66].

These findings confirm that HIF-1α plays an important role in the regulation of ferroptosis. Under hypoxic conditions, HIF-1α can produce antioxidants to resist oxidative stress caused by radiation-induced ROS. It has been confirmed that IR can also cause significant upregulation of HIF-1α, thus further aggravating radiation resistance of hypoxic cells [67,68]. Therefore, HIF-1α is an effective target for radiation sensitization of hypoxic cells [69,70]. Ferroptosis accounts for a large proportion of IR-induced cell death, even more than apoptosis and necrosis [19]. HIF-1α-mediated ferroptosis inhibition is closely associated with poor prognosis and treatment tolerance of hypoxic tumors [60]. Consequently, it may be practical to reduce HIF-1α-induced radiation resistance by regulating the occurrence of ferroptosis.

### 3.2. HIF-2α—A Positive Regulator of Ferroptosis

As two important members of the HIF family, both HIF-1α and HIF-2α share 48% structural homology [71]. Thus, they have certain functional similarities in regulating physiologic and pathologic responses in hypoxic environments and in affecting the development of hypoxia-related diseases. However, increasing evidence has revealed that when expressed in the same cell type, HIF-1α and HIF-2α activation can produce highly different or even opposite results [72,73]. Previous studies on ferroptosis have shown that HIF-2α plays a positive role in ferroptosis in various tumor models [74,75].

Zou et al. showed that HIF-2α activation stimulated hypoxia-induced expression of lipid droplet-associated proteins (HILPDA) and selectively enriched polyunsaturated lipids to improve the sensitivity of clear-cell carcinoma cells to ferroptosis. Notably, HIF-1α also plays a role in increasing ferroptosis sensitivity in ovarian clear cell carcinoma cells [74]. The clear-cell carcinomas (CCCs) are always associated with highly active lipid and glycogen synthesis and deposition, which promotes tumor progression and treatment resistance [76]. HIF-mediated ferroptosis susceptibility may be related to the special metabolic state and high GPX4 dependence of clear-cell carcinoma cells [77]. Therefore, the relationship between HIF and ferroptosis in other tumors with high metabolic activities and GPX4 dependence is worth exploring. In addition, under hypoxic conditions, HIF-2α is a major regulator of erythropoiesis and cellular iron metabolism, which can interfere with bone morphogenetic protein (BMP) signaling, inhibit the expression of hepcidin, and regulate iron uptake and mobilization in the intestinal diet [78,79]. Studies have shown that HIF-2α activation can upregulate the expression of lipid and iron regulation genes as well as increase the intracellular iron level, thereby leading to the susceptibility of colorectal cancer cells to ferroptosis. Furthermore, HIF-2α activation enhances colon cancer cell death by promoting irreversible cysteine oxidation in an iron-dependent manner to increase ROS production. In contrast, HIF-1α has no significant regulatory effect on colorectal cancers [75]. A recent study showed that HIF-2α also mediates ferroptosis in chondrocytes [80], implying a more general role of HIF-2α in ferroptosis in other contexts. However, as a hypoxia-triggered factor, HIF-2α has a similar effect as HIF-1α on radiation resistance, so the role of HIF-2α-mediated ferroptosis in radiotherapy is open to debate.

Radiation and chemotherapy resistance caused by hypoxia has always been a major problem in tumor therapy; however, targeting hypoxic cells remains a challenge, as frequent use of HIF inhibitors can rapidly develop drug resistance in tumor cells. Recent studies have shown that regulating the activity of HIF to promote ferroptosis sensitivity may be a good way to solve the poor prognosis of hypoxic tumors [60]. As mentioned above, HIF-1α inhibits ferroptosis by targeting SLC7A11, lipid metabolism, and iron homeostasis. HIF-2α induces ferroptosis, perhaps because it is a major regulator of iron metabolism in hypoxic conditions. However, both HIF-1α and HIF-2α show promoting effects on ferroptosis in CCCs, suggesting the regulation of ferroptosis by HIF is complex and can be affected by many factors such as tumor types, metabolic levels and external environment. Therefore, further studies on HIF and ferroptosis should be carried out to provide more effective methods for the radiosensitization of solid tumors.

## 4. Oxidative Stress and Ferroptosis in Hypoxic Cells

Oxidative stress (OS) was first proposed in 1970 by Paniker, who studied the role of glutathione reductase in the pentose phosphate pathway [81]. Under normal physiologic conditions, active substances such as ROS and superoxide anions are mostly eliminated by superoxide dismutase (SOD), vitamin E, and other antioxidants [82]. ROS can be converted to H_2_O_2_ under the catalytic action of superoxide dismutase (SOD) [83]. GSH is a key regulator of the antioxidant system, which can reduce H_2_O_2_ to H_2_O and oxidize itself to oxidized glutathione (GSSG) under the action of glutathione peroxidase [84]. The pentose phosphate pathway (PPP) is the first branch of glycolysis, using glucose-6-phosphate as the main substrate. PPP is the main source of NADPH, which mediates the reduction in GSSG and the regeneration of GSH [85]. Hypoxia activates the antioxidant system to protect tumor cells from oxidative stress and thus causes radiation resistance [3]. ROS overload is the basis of ferroptosis. It is widely believed that ferroptosis can kill cells by amplifying oxidative stress or suppressing the antioxidant system [86]. Therefore, we used nuclear factor erythroid 2-related factor 2 (Nrf-2) and AMP-activated protein kinase (AMPK), which are important regulatory factors of oxidative stress, as examples to summarize their roles in ferroptosis and elucidate that hypoxia-induced antioxidant system activation also inhibits ferroptosis.

### 4.1. Regulation of Ferroptosis in Hypoxic Cells by Nrf-2

**Figure 3:**
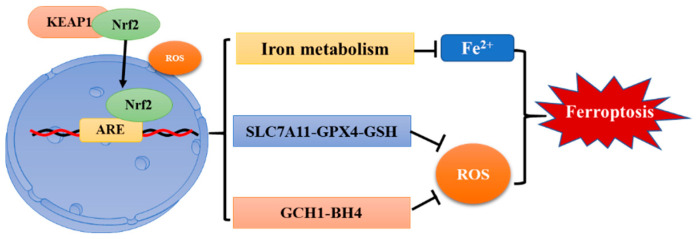
Regulation of ferroptosis by Nrf-2.

The Keap1-Nrf-2-ARE pathway is a classic anti-oxidative stress pathway. Under normal circumstances, Nrf-2 binds to kelch-like ECH-associated protein-1 (Keap1) and exists in the cytoplasm in an inactive state. When stimulated by ROS, Nrf-2 dissociates from Keap1 and transfers to the nucleus, binds with antioxidant-responsive element (ARE) to activate transcription of downstream genes, and then translates a series of related proteins to play physiological functions [87]. Previous studies revealed that Nrf-2 is closely related to hypoxia [88,89]. Hypoxia activates the Keap1-Nrf-2-ARE pathway to protect cells from oxidative damage caused by ROS overload. Moreover, Nrf-2 is positively correlated with HIF-1α. Nrf-2 silence blocks the accumulation of HIF-1α in hypoxic cancer cells, weakens its regulatory effect of cell metabolism, and causes an imbalance in ROS homeostasis [90]. Furthermore, it been reported that Nrf-2 also plays an important role in ferroptosis (Figure 3).

Firstly, Nrf-2 plays a key role in iron metabolism [79]. Both the light and heavy ferritin chains (FTL/FTH1) and ferroportin (SLC40A1), which are responsible for iron transport out of cells, are controlled by Nrf-2 [91,92]. Nrf-2 can positively regulate the transcription of heme-oxygenase 1 (HMOX1); at the same time, Nrf-2 can also rapidly up-regulate the transcription of FLH and FTH to increase iron storage and reduce intracellular free iron, thereby suppressing the occurrence of ferroptosis [93]. Second, Nrf-2 can also induce ferroptosis by promoting the expression of SLC7A11, glutamate cysteine ligase (GCLC/GLCM), and glutathione synthetase (GSS) to mediate glutathione synthesis [94,95,96]. In addition, Nrf-2 directly promotes the expression of GPX4 and regulates the GCH1/BH4 pathway to mediate the redox reaction of cells and inhibit the occurrence of ferroptosis [97,98]. These results demonstrate that Nrf-2 is a key mediator of oxidative stress and ferroptosis.

As mentioned above, HIF-1α enhances the activity of glycolysis, serine synthesis, and pentose phosphate pathways, thereby increasing antioxidant production, buffering radiation-induced ROS, and reducing cellular ferroptosis and radiation sensitivity. It has been reported that Nrf-2 is also involved in HIF-1α-mediated inhibition of ferroptosis. Li et al. demonstrated that iASPP, an inhibitor of apoptosis-stimulating protein of p53, can activate the Nrf-2 /HIF-1α/TF signaling pathway to reduce acute lung injury (ALI) and ischemia/reperfusion-induced ferroptosis [99]. Gourmet et al. also noted that melatonin ameliorates hypoxic-ischemic brain injury by upregulating Nrf-2 to inhibit ferroptosis [97]. Interestingly, Nrf-2 has also been found to be closely related to cellular radiation resistance [100]; silencing Nrf-2 can significantly increase cellular radiation sensitivity, which is still applicable in the treatment of solid hypoxic tumors. For example, Yun et al. demonstrated that the silencing of Nrf-2 can upregulate the expression of human carbon-based reductase 1 (CBR1) at the transcriptional level to improve the radiosensitivity of head and neck tumors [101]. A study in patients with esophageal cancer also showed that high expression of Nrf-2 and SLC7A11 was associated with reduced radiotherapy-induced ferroptosis and lipid oxidation, ultimately leading to radiation resistance [48].

In conclusion, Nrf-2 plays an important role in ferroptosis and radiosensitivity of hypoxic cells. Silencing Nrf-2 may be an effective way to promote ferroptosis of hypoxic cells, thus achieving radiotherapy sensitization of hypoxic tumors.

Nrf-2 is a major regulator of antioxidant response. When Nrf-2 is stimulated by ROS, it dissociates from Keap1 and is transferred to the nucleus, where it binds to ARE and activates the transcription of downstream genes. Nrf-2 can inhibit ferroptosis by regulating proteins related to iron metabolism and ROS clearance pathways. Nrf-2, nuclear factor erythroid 2-related factor 2; Keap1, kelch-like ECH-associated protein-1; ARE, antioxidant-responsive element.

### 4.2. Regulation of Ferroptosis in Hypoxic Cells by AMPK

**Figure 4:**
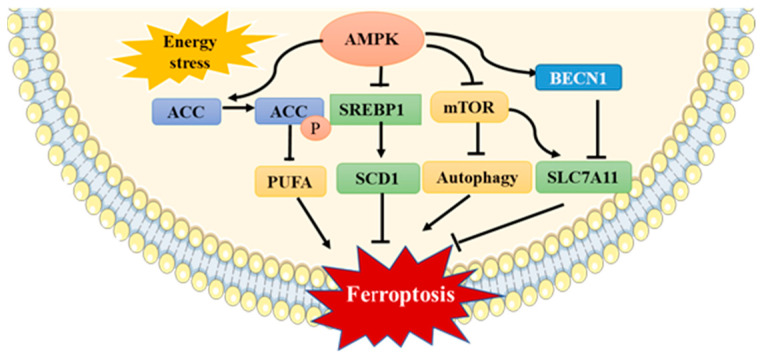
Regulatory effect of AMPK on ferroptosis.

The role of AMPK in substance and energy metabolism has been confirmed. However, studies have found that AMPK can also be activated by oxidative stress to participate in the regulation of the body’s antioxidant system [102]. Increased ROS levels induced by oxidative stress are considered as the main cause of AMPK activation [103]. In recent years, with the deepening of studies on ferroptosis, AMPK has been found to regulate the occurrence and development of ferroptosis as well as oxidative stress [104,105,106,107,108]. However, current studies on AMPK and ferroptosis have produced different or even opposite results, depending on the cell type and treatment conditions. Despite this, the results demonstrated that AMPK is an important target for regulating cellular ferroptosis (Figure 4).

AMPK regulates ferroptosis mainly through the following pathways: energy stress-mediated activation of AMPK inhibits fatty acid synthesis by inactivating acetyl-CoA carboxylase (ACC), thereby reducing ferroptosis sensitivity [104,105]. In contrast, activation of the AMPK-mTOR pathway can reduce the phosphorylation level of the downstream target protein P70S6K, inhibit the expression of SLC7A11, and promote ferroptosis [106]. The AMPK-mTOR pathway has also been shown to induce ferroptosis by promoting autophagy [107]. In addition, a study in hepatoma cells confirmed that AMPK activation downregulated the expression of sterol regulatory element binding protein 1 (SREBP1) and its downstream gene, stearoyl-CoA desaturase-1 (SCD1), as well as reduced the synthesis of mono-unsaturated fatty acids and promoted the occurrence of ferroptosis [109]. Moreover, AMPK-mediated phosphorylation of Beclin 1 (BECN1) induces ferroptosis by directly blocking system X_c_^−^ activity [108].

AMPK can be activated by different molecular mechanisms in different tissues and cell types under hypoxic conditions [110]. Among them, the LKB1-AMPK axis is considered to be the main pathway; the hypomorphic expression of live kinase B1 (LKB1) can inhibit AMPK activation [111]. Additionally, hypoxia is accompanied by a large amount of ROS production. It has been reported that hypoxia-induced ROS accumulation damages mitochondria, inhibits ATP synthesis, and increases AMP/ATP in cells, which may also be a mechanism of AMPK activation [112]. In a diabetic rat and typical energy stress model, it has been confirmed that the activation of AMPK in hypoxic cardiomyocytes inhibits NADPH oxidases 2 (NOX2)-related oxidative stress, thus inhibiting cellular ferroptosis [113]. Although whether other pathways involved in the regulation of ferroptosis by AMPK are applicable in hypoxic cells and whether they exhibit different results in various cell lines remain to be explored, existing studies suggest that AMPK is closely related to oxidative stress, hypoxia, and ferroptosis. It is a key factor in the regulation of ferroptosis in hypoxic cells.

IR can ionize the cytoplasm and mitochondria of tumor cells and produce lots of ROS, which will act on biological macromolecules to form irreversible damage and cause cell death. Previous studies proved that the increase in intracellular ROS content can improve radiosensitivity through inducing autophagy, apoptosis, oxidative stress and other pathways [114,115,116]. However, more and more studies suggest that ferroptosis is also involved in ROS-induced radiosensitization [117,118]. Oxidative stress and hypoxia lead to a large accumulation of ROS. When such accumulation exceeds the regulatory capacity of the cell’s antioxidant system, excessive ROS act on the plasma membrane and cause lipid peroxidation of membrane phospholipids, thereby leading to ferroptosis. However, hypoxia also triggers the activation of antioxidant systems that protect cells from ferroptosis. Both Nrf-2 and AMPK are important regulatory factors of oxidative stress, which can be activated under hypoxia. Studies have demonstrated that they are also involved in the ferroptosis inhibition and radiation resistance mediated by hypoxia as antioxidants. Therefore, breaking intracellular antioxidant systems may be an effective way to induce ferroptosis and enhance the radiosensitivity of hypoxic cells. However, is oxidative stress and ferroptosis synergistic, or is ferroptosis a special form of oxidative stress? Is ferroptosis a novel way to explain other diseases caused by oxidative stress, such as rheumatoid disease? There are no clear answers to these questions; thus, we need to explore them further.

AMPK regulates ferroptosis mainly through the following pathways: activation of AMPK caused by energy stress can lead to phosphorylation and inactivation of ACC, reduce the synthesis of PUFA, and inhibit ferroptosis. Furthermore, AMPK can induce ferroptosis by regulating the activation of the AMPK-mTOR pathway, the inhibition of SCD1, and the phosphorylation of BECN1. AMPK, AMP-activated protein kinase; ACC, acetyl-CoA carboxylase; PUFA, polyunsaturated fatty acids; mTOR, mechanistic target of rapamycin; SCD1, stearoyl-CoA desaturase 1; BECN1, Beclin 1.

## 5. Radiosensitization of Hypoxic Cells Mediated by Ferroptosis

Radiotherapy (RT) is a treatment that uses high-energy radiation to locally control many types of tumors. Hypoxia is a common feature of solid tumors, which is caused by an imbalance between oxygen supply and consumption. It is also considered as one of the most important reasons for radiotherapy failure [119,120]. After years of exploration, a number of strategies have been developed to overcome radiation resistance from hypoxia, including improving tumor oxygenation through hyperbaric oxygen, cytotoxin, and oxygen-mimicking radiosensitizers [121]. However, their clinical use has been limited by their side effects and poor efficacy to date.

With the deepening of research, it was found that ferroptosis accounted for a large proportion of cell death caused by radiation [19]; the radiation sensitization effect of ferroptosis inducers (FINs) has gradually gained attention (Table 1). Lang et al. used FINs, such as sulfasalazine, RSL3, and Cyst(e)inase, to treat cells; they found that FINs had a significant radiosensitizing effect on HT1080 cells and B16F10 cells in vitro and in vivo [18]. Lei et al. applied class I, II, and III FINs (Table 2) to non-small cell lung cancer cells and found that they all showed significant radiosensitizing properties. Their sensitization effect is even stronger than those inducing apoptosis, necrosis, and other traditional cell death modes [19]. Moreover, Ye et al. demonstrated that radiation combined with IKE or sorafenib inhibited tumor growth in mouse xenograft models [122].

Hypoxia inhibits ferroptosis by suppressing ferritinophagy, activating the HIF-1/2α pathway and facilitating the antioxidant system, resulting in poor prognosis and radiation resistance of tumors. Hence, FINs and radiosensitizers may overlap in hypoxic cells under certain conditions.

Nitroimidazole is a radiosensitizer that is commonly used in clinics. Under hypoxic conditions, nitroimidazole can replace oxygen as an electron acceptor, thus enhancing the effect of radiation on malignant cells [123]. Recent studies have demonstrated that the radiosensitization effect of 2-nitroimidazole on glioma stem cells under hypoxia is ferroptosis-dependent. This can cause ferroptosis in several ways. First, the activity of mitochondrial complexes I and II decrease, whereas the ratio of NADH/NAD+ increases. These processes lead to the accumulation of ROS, which is more obvious under hypoxia. In addition, 2-nitroimidazole can also cause the upregulation of Steap3 and HMOX1, leading to an increase in iron in the labile iron pool to induce ferroptosis, which directly leads to the radiosensitization of hypoxic GSCs [124]. Other than addition, a study on triple-negative breast cancer also showed that iron-saturated Lf (Holo-lactoferrin, Holo-Lf) can improve the radiotherapy effect of MDA-MB-231 cells in a hypoxic environment by influencing HIF-1α expression and ROS production to induce ferroptosis [21].

Many antineoplastic drugs can also act as FINs. Alkylating agents are classical chemotherapy drugs, which can cause ROS accumulation and oxidative stress in tumor cells such as radiotherapy. Cisplatin is a typical example, it can trigger ferroptosis by directly consuming intracellular GSH and inhibiting GPX4 [125]. Temozolomide (TMZ) is another alkylating agent, which induces ferroptosis by promoting SLC7A11 expression at mRNA and protein levels through Nrf-2 and activating transcription factor 4 (ATF4) activation pathways [126]. In addition, sorafenib, a molecular-target drug, can inhibit System X_C_^−^, leading to endoplasmic reticulum stress and GSH depletion, thereby facilitating ferroptosis [127]. Furthermore, it can act on the HIF-1α/SLC7A11 pathway to promote ferroptosis [128]. Radiotherapy combined with antitumor drugs has been widely used in clinical practice. Cisplatin combined with radiotherapy significantly prolonged the survival time and improved the prognosis of patients with head and neck squamous cell carcinoma, non-small cell lung cancer, cervical cancer, vulva squamous cell carcinoma and other cancers [129,130,131,132,133]. The combination of sorafenib with temozolomide and radiotherapy observably reduced glioblastoma multiforme cell viability compared to radiotherapy alone [134]. However, whether ferroptosis plays a role in this combination therapy is unclear. Due to the short discovery time of ferroptosis, people’s understanding of its mechanism is not perfect. In addition, some FINs are poorly tolerated in vivo or even have toxic side effects; for example, animal experiments found GPX4 knockout causes embryonic lethality in mice [135]. Although more and more evidence has proven that ferroptosis is closely related to radiotherapy, there are still many aspects to be explored before FINs are really applied to the clinic.

**Table 1:** Radiosensitizing effect of ferroptosis inducers on tumor cells.

FINs	Cancer Type	Mechanism	Reference
Erastin	Breast cancer	Inhibit system X_c_^−^ and prolong the duration of radiation-induced DNA damage	[136]
	Cervical cancer	Reduce glutathione concentration	[137]
	Lung cancer	Inhibit the expression of GPX4	[138]
Sulfasalazine	Melanoma	Decreases the level of GSH	[139]
	Glioblastomas	Reduced cystine uptake and GSH level	[140]
Gallic acid	Breast cancer	Decrease GPX4 activity	[141]
	Melanoma		
IKE/RSL3/sorafenib	Sarcoma	Enhance lipid peroxidation	[122]
ML162/FIN56	Lung cancer	Degrade GPX4 and deplete antioxidant CoQ10	[19]

GPX4, glutathione peroxidase 4; GSH, glutathione; RSL3, Ras-selective lethal small molecule 3; IKE, imidazole ketone erastin; COQ10, coenzyme Q10.

**Table 2:** Several common ferroptosis inducers and their mechanism of action.

Class	Drugs	Mechanism
Class I	Erastin/PE/IKE, Sulfasalazine, Sorafenib, BSO, DPI2, Cisplatin, Cyst(e)inase	Inhibit system X_c_^−^; Prevent cystine import
Class II	RSL3, (1S, 3R)-RSL3, DPI7 (ML162), DPI10 (ML210)	Inactivate GPX4
Class III	FIN56, Statins	Degrade GPX4; Deplete antioxidant CoQ10
Class IV	Artemisinin, FINO2	Impair IRP/IRE; Inactivate GPX4 indirectly

IKE, imidazole ketone erastin; BSO, buthionine sulfoximine; RSL3, Ras-selective lethal small molecule 3; GPX4, glutathione peroxidase 4; COQ10, coenzyme Q10; IRP/IRE, iron-regulatory protein/iron-responsive element.

## 6. Conclusions and Perspectives

Radiation therapy can cause DNA strand breakage, membrane rupture, and other reactions in tumor cells, ultimately leading to death. Currently, it is increasingly being applied in tumor treatment. However, radiation resistance caused by hypoxia is still an important factor that limits the effect of radiotherapy. At present, many methods have been explored to improve the oxygenation ability of hypoxic cells in order to potentiate radiotherapy; however, the consequences are not satisfactory. In recent years, ferroptosis is considered as a newly discovered mode of cell death. Hypoxia can inhibit ferritinophagy, reduce intracellular free iron, and avoid ferroptosis, which may be a potential mechanism leading to radiation resistance in hypoxic cells. HIF-1/2α is also involved in the regulation of ferroptosis under hypoxic conditions via complex pathways. Inducing ferroptosis by controlling iron metabolism and HIF-1/2α downstream pathways may be an effective method to enhance the radiosensitivity of hypoxic tumor cells. In addition, ferroptosis is often accompanied by oxidative stress. ROS overload and lipid peroxidation are basic characteristics of it. In fact, there is evidence that hypoxic cells are highly dependent on their antioxidant systems. Therefore, promoting ferroptosis to upregulate ROS in cells or inhibit their antioxidant system activity may be a feasible radiosensitization method. In conclusion, with the emergence of new insights into the interaction between radiation, hypoxia, and ferroptosis in the fight against hypoxia-induced radioresistance, alleviating hypoxia-induced ferroptosis inhibition may be safe and efficient as a radiosensitizing method and deserves more attention.
